# Transfer of remote ischemic preconditioning plasma from heart transplant patients into isolated perfused rat hearts prior ischemia/reperfusion injury—a translational study of cardioprotection

**DOI:** 10.3389/fcvm.2025.1631222

**Published:** 2025-08-29

**Authors:** Gizem Bingöl, Steve Jesurasa, Martin Stroethoff, Julian David Jagdfeld, Leila Sophie Henning, Sebastian Roth, Giovanna Lurati Buse, René M'Pembele, Annika Raupach

**Affiliations:** ^1^ Department of Anaesthesiology, Medical Faculty, University Hospital Düsseldorf, Heinrich-Heine-University Düsseldorf, Düsseldorf, Germany; ^2^ CARID, Cardiovascular Research Institute Düsseldorf, University Hospital Düsseldorf, Heinrich-Heine University Düsseldorf, Düsseldorf, Germany

**Keywords:** Langendorff, cardioprotection, plasma transfer, infarct size, heart transplantation, cardiac surgery, translational study

## Abstract

Remote ischemic preconditioning (RIPC) has been shown in several experimental studies as an organ protective procedure against ischemic injury, but the implementation of RIPC into routine clinical practice has so far failed due to contradictory study results. However, in order to identify patient groups that could benefit from RIPC, numerous clinical trials have been initiated, but only one study with patients undergoing heart transplantation (HTX). In HTX patients, RIPC appears to be cardioprotective when used immediately before surgery, while it has not been investigated whether the cardioprotective effect of RIPC is longer lasting. Therefore, this study assessed if a RIPC procedure prior to HTX has a cardioprotective potential in a later time window. To avoid masking a potential cardioprotective effect of RIPC in HTX patients by reduced susceptibility to cardioprotective signals due to comorbidities and medications in these patients, this study investigates the protective potential of this plasma in healthy young rats. Thus, male HTX patients were treated with a sham or a RIPC procedure (3 cycles with 5 min inflating/deflating) via a blood pressure cuff at the left upper limb prior surgery. After HTX, blood was collected at arrival on intensive care unit, 24 and 48 h post-surgery. The isolated plasma was transferred to isolated-perfused rat hearts before induction of ischemia/reperfusion injury. Cardiac function was determined by left ventricular pressure measurements and infarct size by triphenyltetrazolium chloride staining. In all measurements, no differences were observed between the sham- or RIPC plasma-treated groups at the respective time points. This suggests that RIPC plasma from HTX patients, at least in the experimental setup used, has no cardioprotective potential at later time points. This lack of effect could for instance be explained by either no or an insufficient amount of cardioprotective signals are produced or/and released into the blood following the RIPC procedure and needs to be explored in future studies.

## 1 Introduction

Remote ischemic preconditioning (RIPC) has been shown in several experimental studies as a protective procedure against ischemic injury in several organs, such as the heart or the kidneys (1, 2). The RIPC procedure consists of inflating and deflating a blood pressure cuff on a distal limb for usually three cycles of five minutes each. This technique is particularly promising for transfer from bench to beside, as it is simple, non-invasive, cost-effective, and easily performed by clinical staff. However, the introduction of RIPC into routine clinical practice has so far failed due to confounding factors of cardioprotection such as age, comorbidities and medications, which are probably responsible for contradicting efficacy study results (2). Nevertheless, in order to identify patient groups that could benefit from a protective effect of RIPC, numerous clinical studies were conducted with patient groups undergoing various surgical procedures. For example, a meta-analysis that examined 72 randomized controlled trials indicates that RIPC can improve survival, reduce postoperative stroke, and shorten hospital stays for non-cardiac surgeries (3).

The underlying mechanism of the organ protective effect by RIPC is not yet fully understood (2), but there is consensus, that the remote organ/tissue generates signals, which are transferred in the effector organ and induce cardioprotective signaling (4). These signals can be humoral, neuronal or systemic triggers and pathways, and the relevance and degree of interaction between these three systems may vary depending on conditioning stimulus. In contrast to ischemic preconditioning (IPC) mediating protection in two windows (early/acute (<4 h after conditioning) and a late/delayed (24–72 h after conditioning), RIPC seems to confer protection in a more continuous and long-lasting manner (5), whereas studies also show a discontinuous protection by RIPC (6, 7). Directly after the conditioning stimulus, protection is achieved through the rapid activation of signaling cascades and the release of already produced humoral factors into the bloodstream. In the later phases, the released humoral factors and modulated pathways seems to be the result of new protein synthesis (8). Due to the release of humoral factors such as specific microRNAs or nitrite in the blood, the protective effect of RIPC is transferable between species by plasma transfer (9–12). For example, human RIPC plasma from young, healthy men transferred to isolated-perfused rat hearts is able to reduce infarct size after ischemia/reperfusion (I/R) injury (13). For the transfer of human RIPC plasma to hearts from rodents in an I/R injury model, a continuous protective potential by reduction of infarct size is shown, with a beginning immediately after RICP and sustained till 6–9 days (14, 15). In these studies, only young and healthy volunteers were used, whereas a kinetic of transferred RICP plasma in patients is not demonstrated yet. Studies have already shown that transferred RIPC plasma from patients undergoing cardiac surgeries can be cardioprotective in the rodent heart, but these studies only collected plasma immediately or 30 min after RIPC and did not examine plasma at later collection time points (16, 17).

Organ-protective effects of RIPC have also been demonstrated in the context of cardiac surgeries, as highlighted by meta-analysis reporting a reduced incidence of acute kidney injury (AKI) (18, 19). The included studies mainly investigated procedures such as coronary artery bypass grafting (CABG) and aortic valve replacement (AVR). In contrast, for patients undergoing heart transplantation (HTX), only one study has examined the potential protective effects of RIPC—a small clinical trial with 60 patients in each treatment group (20). In this study, RIPC was performed directly after anesthesia for HTX in combination with remote ischemic postconditioning (RIPostC) 20 min after aortic declamping. The authors observed a reduction in serum levels of the myocardial injury marker cardiac troponin I (cTnI) at 6 h after aortic declamping, whereas no improvement in clinical outcomes was noted (20). Thus, RIPC was been shown to mitigate I/R damage during the acute window in HTX patients, whereas a cardioprotective effect by RIPC in such patients in later phases has not yet been investigated separately.

Therefore, the aim of this study is to assess if a RIPC procedure prior to HTX has a protective potential against oxidative stress like I/R injury in the later phases and provides long-lasting protection. To avoid the possibility that a potential cardioprotective effect of RIPC in HTX patient is masked by a reduced responsiveness to cardioprotective signaling due to comorbidities and medications in such patients, this study investigates the protective potential of this plasma in healthy young rats. Therefore, plasma from male HTX patients treated with a sham or a RIPC procedure was collected at three different time points after HTX and transferred to isolated-perfused rat hearts to examine a cardioprotective effect against I/R injury.

## 2 Materials and methods

### 2.1 Animals

All experiments were performed in accordance with the Guide for the Care and Use of Laboratory Animals, published by the U.S. National Institute of Health (NIH publication No. 85-23, revised 1996), after approval by the Central Institution for Animal Research and Scientific Animal Welfare (ZETT) Heinrich-Heine University, Düsseldorf, Germany (project number O27/12). Male Wistar rats aged 2–3 months were used in this study. The rats had *ad libitum* access to food and water during a 12-hour light-dark cycle. In this study, 56 rats were included.

### 2.2 Clinical study and plasma sampling

**Figure 1:**
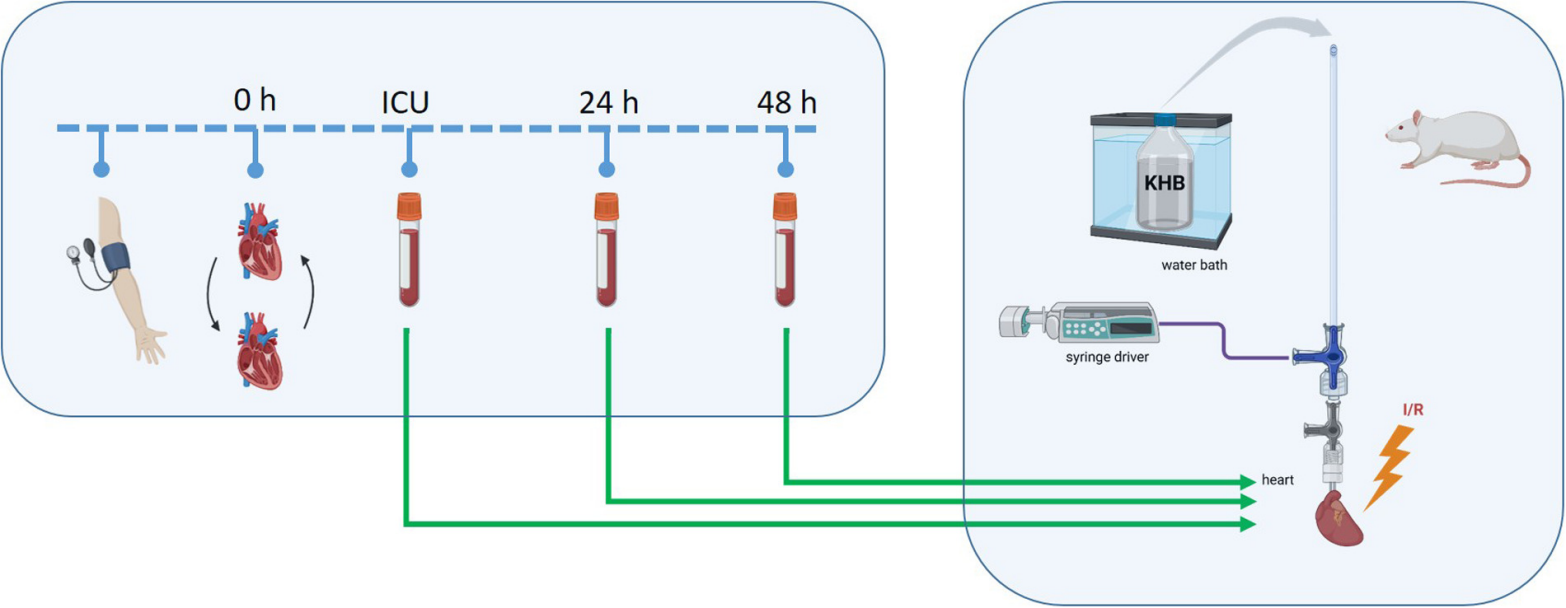
Study scheme. Before heart transplantation (0 h), patients underwent either a sham or remote ischemic preconditioning (RIPC) procedure using blood pressure cuffs. Blood samples were collected upon intensive care unit (ICU) arrival after surgery and at 24 h and 48 h post-surgery, followed by plasma isolation. The obtained plasma was then transferred via a syringe driver to isolated-perfused rat hearts using the Langendorff system to assess its cardioprotective potential against ischemia/reperfusion (I/R) injury. KHB, Krebs-Henseleit buffer. Created in https://BioRender.com.

Plasma for this study was obtained from the RIPCAT clinical trial (**Effect of Remote Ischemic Preconditioning on Acute Kidney Injury in Patients Undergoing Heart Transplantation—A Randomized Controlled Trial**) (NCT05364333). The use of plasma for the present study was approved by the Ethics Committee of Heinrich-Heine University, Germany with an amendment (Study Number: 202-1240_3; 17.07.2024). The clinical trial included adult patients undergoing HTX at the University Hospital Düsseldorf (Figure 1). Key exclusion criteria were: acute myocardial infarction within 7 days before surgery, age under 18 years, pre-existing AKI, previous kidney transplantation, chronic kidney disease with a glomerular filtration rate below 30 ml/min, pregnancy, peripheral vascular disease affecting the upper limbs, hepatorenal syndrome, and ongoing drug therapy with sulfonamides or nicorandil (preconditioning-blocking and preconditioning-mimetic medications, respectively). Patients were randomized into their respective groups before surgery after eligibility was confirmed and written informed consent was obtained. For the present study, only male patients were selected. General anesthesia was induced with etomidate and sufentanil and maintained with sevoflurane and sufentanil during the surgical procedure. The study did not control for postoperative sedation and this was conducted according to local standards and physician's choice. After induction of anesthesia for HTX, patients were treated as following. Patients in the RIPC group were treated with 3 cycles of inflating and deflating with a blood pressure cuff of the left upper limb. The cuff was inflated at 200 mmHg for 5 min (or at least 50 mmHg above the systolic arterial blood pressure) followed by deflating for 5 min allowing reperfusion. In previous studies RIPC cycle-durations of 5 min showed protective effects against development of AKI (21). For sham treatment, the cuff was inflated to 20 mmHg for 5 min followed by 5 min of deflation. Blood samples were collected after HTX surgery upon intensive care unit (ICU) arrival and at 24 and 48 h postoperatively, so the two later sampling times should be within the known delayed protection window of 24–72 h from IPC (4). Blood was drawn from arterial catheters into EDTA vials and plasma isolated (centrifugation: 10 min, 1,000 × g, 4°C). The resulting plasma was stored at −80°C. For characterization of patients, plasma levels of high-sensitive Troponin-T was determined.

### 2.3 Langendorff system

Rats were randomly assigned into the respective groups. Sedation was induced by intraperitoneal injection of 100 mg/kg body weight sodium-pentobarbital (Narcoren, Boehringer Ingelheim, Germany). To prevent agglutination, 3,330 IU/kg heparin sodium (Braun SE, Melsungen, Germany) was injected. After sufficient sedation depth, indicated by a loss of toe pinch reflex, rats were decapitated. Then the chest was opened, heart was isolated, mounted on a constant pressure Langendorff system (80 mmHg), and perfused with a modified Krebs-Henseleit buffer (KHB; 118 mM NaCl, 4.7 mM KCl, 1.2 mM MgSO4, 1.2 mM KH2PO4, 24.9 mM NaHCO3, 2.5 mM CaCl2, 1 mM lactate, 0.5 mM EDTA; and 11 mM glucose at 37°C). Global ischemia was induced by interrupting KHB perfusion while immersing the heart in nitrogen-aerated KHB. Reperfusion was initiated by restoring perfusion with KHB while simultaneously removing the ischemic buffer bath. The plasma was introduced into the perfusate via a syringe driver with 1% of the coronary flow (CF). This plasma concentration had been shown in own previous studies to be cardioprotective by reducing infarct size when RIPC plasma from healthy subjects was transferred to rat hearts after I/R injury (13, 22). CF was determined by collecting effluent over 1 min. For continuous left ventricle (LV) pressure measurements, a water-filled balloon was placed in the LV, and the pressure changes were digitized using an analog to digital converter (PowerLab/8SP, ADInstruments Pty Ltd, Castle Hill, Australia) at a sampling rate of 500 Hz. Data were recorded on a personal computer using Labchart 8.0 for Windows (ADInstruments Pty Ltd, Castle Hill, Australia). During the adaptation phase, the left ventricular diastolic pressure (LVP min) was set at 3–5 mmHg. The following hemodynamic variables were determined from pressure measurements of the balloon placed in the LV: heart rate, LVP min, left ventricular systolic pressure (LVP max), left ventricular developed pressure (LVDP = LVP max—LVP min), maximum rate of pressure change in the LV (dP/dt max), and minimum rate of pressure change in the LV (dP/dt min).

### 2.4 Experimental setup

**Figure 2:**
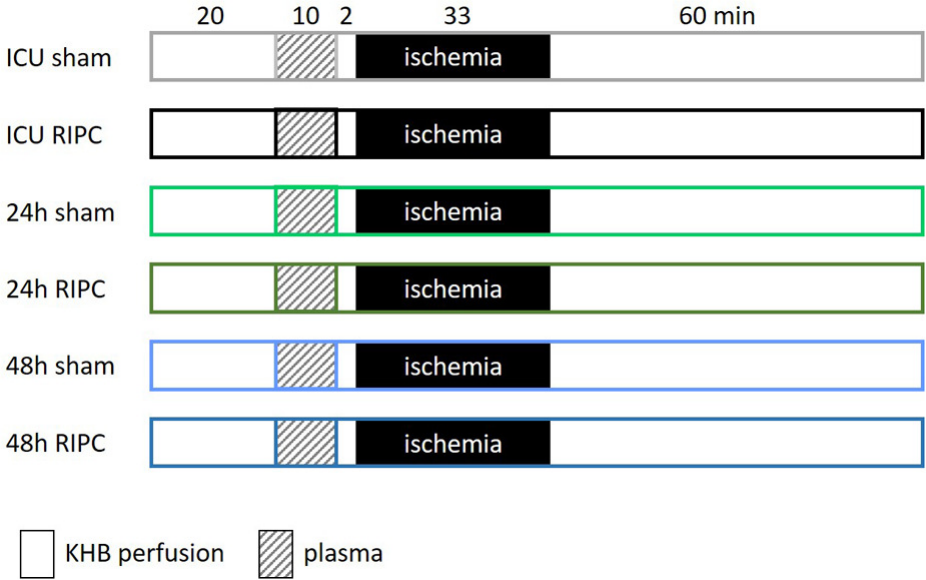
Experimental protocol: after 20 min of adaption, hearts of male Wistar rats are perfused with the respective plasma for 10 min followed by a 2 min wash out phase. Afterwards, the hearts are subjected to 33 min ischemia and 60 min reperfusion. Plasma was isolated from blood of patients who were treated with a sham or a RIPC (remote ischemic preconditioning) procedure prior to heart transplantation. The time points for blood collection were on arrival in the intensive care unit (ICU) after surgery and 24 and 48 h after surgery. Krebs-Henseleit-buffer (KHB).

All hearts underwent the following protocol: 20 min adaption phase, 10 min treatment with plasma, 2 min washout, 33 min ischemia, and 60 min reperfusion (Figure 2). The plasma treatment duration of 10 min had been shown in own previous studies to be cardioprotective by reducing infarct size when RIPC plasma from healthy subjects was transferred to rat hearts after I/R injury (13, 22). Each heart was treated with only one plasma sample, allowing each sample to be clearly assigned to a specific heart.

### 2.5 Infarct size determination

To assess infarct size, heart slices (1 mm thick) were stained with 0.75% triphenyltetrazolium chloride (TTC, #37130.02, SERVA Electrophoresis GmbH, Heidelberg, Germany). For quantification, an investigator blinded to group assignment measured the total LV area and infarcted area using planimetry with SigmaScan Pro 5 (Systat Software Inc., San Jose, USA). Infarct size was then calculated as the infarcted area expressed as a percentage of the total LV area.

### 2.6 Statistics

An *a priori* sample size analysis was conducted using G*Power 3.1.9.7 (23), with infarct size as the primary endpoint. All other statistical analyses were performed using Prism 10 (GraphPad, Boston, MA, USA). Group characteristics were compared using the *t*-test to assess for statistical differences between the two patient groups. The impact of RIPC on infarct size was analysed using the *t*-test at the respective plasma collection time point; the calculated sample size for detecting a mean difference of 20% with a standard deviation (SD) of 14% in infarct size was 9 per group (power 80%, *α* 0.05, effect size 1.43). For variables measured longitudinally over time, baseline measurements were compared using one-way analysis of variance (ANOVA) to ensure equivalent starting points across groups. In addition, for comprehensive assessment, measurements taken after 60 min of reperfusion were analysed for the respective time point each.

The level of significance was defined as *p* < 0.05, otherwise the effect was declared not significant (ns). Data are presented as mean ± SD.

## 3 Results

### 3.1 Characteristics of patients

The patients randomized to the respective treatment groups—sham or RIPC—did not differ in their characteristics such as age or body mass index or comorbidities including diabetes, coronary heart disease, chronic obstructive pulmonary disease, and arterial hypertension (Table 1). Furthermore, intra- and postoperative severity or damage parameters, including the vasoactive-inotropic score, cardiopulmonary bypass time, and plasma high sensitive Troponin T levels, were comparable between groups, indicating a homogeneous patient cohort.

**Table 1:** Patient characteristics.

Variable	sham	RIPC	*p*
Age (years)	57 ± 9	57 ± 10	0.97
Number of patients	13	13	na
BMI (kg/m^2^)	24.2 ± 3.4	25.5 ± 2.4	0.30
Smoker	9	7	0.44
Diabetes	4	3	0.67
CPB (min)	265 ± 58	240 ± 66	0.32
VIS	21.3 ± 9.7	20.8 ± 12.2	0.92
Troponin T ICU (ng/L)	3,378 ± 1,534	3,515 ± 2,219	0.86
Troponin T 24 h (ng/L)	1,200 ± 1,032	1,760 ± 1,908	0.65
Troponin T 48 h (ng/L)	2,158 ± 2,309	1,225 ± 1,509	0.91
Arterial hypertension	4	8	0.13
CHD	9	7	0.44
COPD	2	4	0.37

Male patients were randomized in the respective groups and were pretreated with a sham or a remote ischemic preconditioning (RIPC) procedure before heart transplantation (HTX). From these patients, blood was collected at three time points: on arrival in the intensive care unit (ICU) after HTX or 24 or 48 h after surgery.

BMI, body mass index; CPB, cardiopulmonary bypass time; VIS, vasoactive inotropic score; CHD, coronary heart disease; COPD, chronic obstructive pulmonary disease. Data are mean ± SD, *n* = 13. T-test revealed no significant differences. *p*, *p*-value, na, not applicable.

### 3.2 Langendorff results

**Figure 3:**
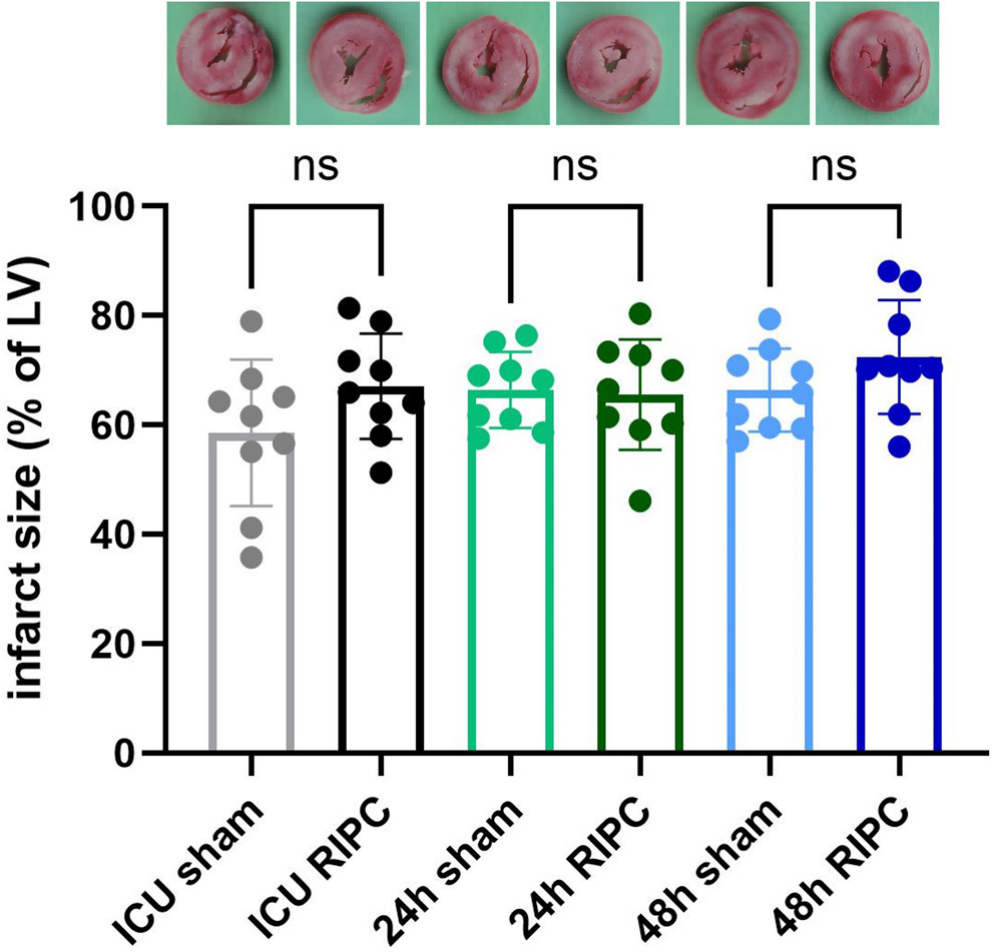
Infarct sizes: quantification of infarct sizes from rat hearts treated with human plasma before ischemia and reperfusion. A representative heart slide stained with triphenyltetrazolium chloride (TTC) is shown for each group. Plasma was isolated from blood of patients pretreated with a sham or a remote ischemic preconditioning (RIPC) procedure before heart transplantation (HTX). The time points for blood collection were: on arrival in the intensive care unit (ICU) after HTX or 24 or 48 h after surgery. LV, left ventricle. Data are mean ± SD, *n* = 9. T-tests at the respective time points of plasma collection revealed no significant differences (ns) between groups sham and RIPC (*p* > 0.05).

The cardioprotective effect of RIPC plasma obtained from HTX patients at different time points after surgery was evaluated in rat hearts subjected to I/R injury. As shown in Figure 3, infarct sizes did not differ between the sham and RIPC groups at any of the assessed time points (ICU sham: 59 ± 13%, ICU RIPC: 67 ± 10%, 24 h sham: 66 ± 10%, 24 h RIPC: 66 ± 10%, 48 h sham: 66 ± 8%, 48 h RIPC: 72 ± 10%; sham vs. RIPC = ns).

No significant differences were observed between groups regarding peak pressure during ischemia (ischemia peak), body weight, or heart weight (both wet and dry) (Table 2). Variables of heart function such as heart rate, LVP min, LVP max, LVDP, dP/dt max, dP/dt min, and CF were comparable at baseline across all six groups, ensuring equal starting conditions (Supplementary Figure S1). Even after 60 min of reperfusion, no differences in these functional variables could be detected between the groups.

**Table 2:** Peak pressure during ischemia (ischemia peak), body and heart weight.

Variable	ICU sham	ICU RIPC	24 h sham	24 h RIPC	48 h sham	48 h RIPC	*p*
Body weight (g)	284 ± 23	281 ± 23	282 ± 20	296 ± 26	286 ± 9	284 ± 9	0.63
Ischemia peaks (mmHg)	74 ± 11	71 ± 8	78 ± 17	82 ± 17	75 ± 14	77 ± 16	0.67
Heart weight wet (mg)	969 ± 73	941 ± 57	978 ± 82	999 ± 91	1,049 ± 58	990 ± 68	0.07
Heart weight dry (mg)	178 ± 19	166 ± 15	172 ± 15	174 ± 12	180 ± 15	179 ± 17	0.39

Data of rat hearts treated with human plasma before ischemia and reperfusion. Plasma was obtained from patients pretreated with a sham or a remote ischemic preconditioning (RIPC) manoeuver before heart transplantation (HTX) and collected at three time points: on arrival in the intensive care unit (ICU) after HTX or 24 or 48 h after surgery. Data are mean ± SD, *n* = 9. One-way ANOVA revealed no significant differences.

*p*: *p*-value.

Taken together, RIPC plasma has no influence on infarct size or heart function compared to sham plasma and this was independent of collection time point.

## 4 Discussion

In this study, we could show that the transfer of RIPC plasma, obtained from HTX patients, to rat hearts has no influence on infarct size or heart function after I/R injury in comparison to sham plasma. This outcome was independent of the time point of plasma collection after HTX surgery. This suggests that, RIPC plasma from HTX patients, at least in the chosen study design, has no cardioprotective potential even in the late phases and that either no, or an insufficient amount of, cardioprotective signals are produced or/and released into the blood following the RIPC procedure.

The absence of cardioprotection observed in this study contrasts with previous studies, in which the transfer of plasma collected 5 min till 9 days after RIPC from healthy volunteers (14, 15) or plasma directly collected after RIPC from patients undergoing a less invasive CABG surgery (16) led to a reduction in infarct size in rodent hearts subjected to ischemia/reperfusion injury. In the following discussion, differences between these three studies and the present study will be explained and several possible explanations for the lack of a protective effect will be considered, including the illness-related burden and characteristics of the patient cohort, experimental setup, and the potential influence of comedications/anesthesia that may have interfered with RIPC-mediated cardioprotection.

One possible explanation for the absence of cardioprotection observed in the present study is that HTX patients are already so severely burdened by their underlying disease and the stress of HTX surgery that no cardioprotective factors are released into the bloodstream. However, a clinical study by Wang et al. reported that in HTX patients, a combination of RIPC and remote ischemic postconditioning (RIPostC) reduced serum levels of cTnI six hours after aortic declamping. Nevertheless, no improvement in clinical outcomes was observed (20). These findings suggest that HTX patients are, in principle, capable of both releasing and responding to cardioprotective signals, but that stronger or combined triggering strategies—such as RIPC with RIPostC—maybe required to achieve measurable effects. The study by Wang et al. is the only study investigating the cardioprotective potential of RIPC in HTX patients so far. It is possible that extending the treatment duration beyond 10 min or increasing the plasma concentration used here by 1% could have compensated for the presumably lower release of cardioprotective factors in this severely ill patient population. However, for RIPC plasma from healthy volunteers transferred to rat hearts, cardioprotective effects on infarct size after I/R injury have already been demonstrated even with shorter treatment durations (8 min) or lower plasma concentrations (0.5% of coronary flow) (10, 13, 22). Thus, the treatment conditions appear to have been sufficient based on studies with healthy volunteers; however, whether these conditions should be adjusted to the specific characteristics of HTX patients remains to be determined by future studies. In principle, however, a cardioprotective effect by RIPC plasma from patients can be induced in isolated perfused hearts, as a study from Lieder et al. demonstrated reduced infarct size after I/R injury in mouse hearts by a treatment with plasma of CABG patients undergoing a less complex surgery (16). In this study, dialyzed plasma collected 30 min after RIPC was transferred for 15 min (dilution 1:10) suggesting that plasma from patients, but most likely not as severely stressed as HTX patients, may be protective at higher concentrations. Therefore, a modification of the experimental setup could be helpful to detect the—if existing—presumably very low protective potential of RICP plasma from critically ill HTX patients.

Moreover, this cohort of HTX patients also suffers from comorbidities such as CHD and arterial hypertension, which—either alone or in combination, as well as through associated medication—may further impair cardioprotective potential (24). For instance, a study by Farkasova et al. (25) demonstrated an inhibitory effect of hypertension on RIPC-induced cardioprotection in aged rats. In this study using a model of isolated-perfused hearts, RIPC on spontaneously hypertensive rats (SHR) led to reduction in infarct size after I/R injury at the ages of 3 and 5 months, whereas no cardioprotection was observed in 8-month-old rats. It should be considered that the HTX patient cohort is also of advanced age and may therefore be subject to similar inhibitory effects. A recent review presents an ambivalent picture regarding the influence of hypertension on conditioning-induced cardioprotection, as studies report both a loss and preservation of protection in the context of hypertension (24). The specific effects of individual comorbidities on RIPC-induced cardioprotection in HTX patients would need to be investigated by subgroup analyses in future studies with a much larger patient cohort.

A further explanation for the absence of cardioprotection in the present study could be the time points of plasma collection. In the case of discontinuous protection by RICP or RIPC plasma, the failing cardioprotection would not have been unexpected at the sampling time point immediately after ICU arrival as it does not fall within the classic protective window of conditioning (4). A loss of the cardioprotective effect of RIPC after an early protective window was shown, for example, by Johnsen et al. (6). These authors could demonstrate that RIPC on the limbs of mice is cardioprotective by reducing myocardial infarct size when applied 0.5 h and 1.5 h before I/R, but loses this effect when applied 2 and 2.5 h before. This observation is consistent with findings by Loukogeorgakis et al., who reported that RIPC failed to provide protection in humans when applied 4 h prior to I/R injury (7). In their study, RIPC attenuated I/R-induced vascular injury, as assessed by flow-mediated dilation, when administered 24 or 48 h before the insult, but not when applied 4 h in advance. However, this would not explain the lack of protection observed with the blood samples taken at 24- and 48 h post-surgery in the present study, as these time points fall within the classical window of delayed protection (24–72 h after conditioning) and therefore a protective effect would have been expected at these times. In the case of continuous protection, protection by the RIPC plasma would have been expected at all time points. Such continuous protection by transfer of RIPC plasma was demonstrated in two studies published by the same group (14, 15). For example, Hildebrandt et al. could show a cardioprotective effect of human RIPC plasma collected 5 min, 30 min, 1 h, 6 h, and daily from 1 to 6 days after the RIPC stimulus, when transferred into a mouse heart before I/R injury (14). In summary, irrespective of potential continuous or discontinuous protection, a reduction in infarct size at least at the collection time points 24 h and 48 h would have been expected. Therefore, the timing of blood sample collection likely played a minor role, and other factors may be more critical in limiting the potential protective effect of RIPC plasma.

However, the lack of protection observed in the present study does not necessarily indicate that plasma from patients with cardiac disease generally lacks organ protective potential. In the case of the acute protection phase, plasma collected from CABG patients 30 min after RIPC reduced infarct size in mouse model of I/R injury (16). At later collection time points, a clinical study demonstrated that an RIPC procedure performed 24 h prior to cardiac surgery reduced the incidence of AKI, indicating that RIPC can exert protective effects on the kidneys within the delayed protection window (26). However, the authors were unable to demonstrate any cardioprotective effects in the patients, which is consistent with the absence of cardioprotection observed in the present study. Perhaps, the cardiac surgery patients in the clinical trial may not have benefited from a cardioprotective effect due to the severity of HTX or presence of comorbidities and medications, which may reduce responsiveness to cardioprotective signaling (27). To circumvent this, the present study used healthy young rats, which would allow to detect also weak cardioprotective effects, which, however, were also absent in this study.

The absence of cardioprotection by RIPC plasma may also be attributed to the patients' anesthesia regimens, which could interfere with cardioprotective signaling (24). For example, all patients received propofol post-surgery. However, it is known that propofol can inhibit cardioprotection, whereas volatile anesthetics such as isoflurane or sevoflurane are considered cardioprotective themselves (28). For instance, in an *in vivo* rat model of I/R injury, RIPC significantly reduced infarct size under pentobarbital and sevoflurane anesthesia, whereas this protective effect was lost under propofol anesthesia (29). In clinical settings, Kottenberg et al. demonstrated that RIPC performed prior to CABG reduced cardiac troponin I levels under isoflurane anesthesia, but not under propofol (30). Nevertheless, the inhibitory role of propofol remains controversial, as meta-analyses investigating the protective effect of RIPC on the incidence of AKI following cardiac surgery have yielded inconsistent conclusions (19, 31). Interestingly, an actual study suggests that the timing of propofol administration may be critical as showing that starting propofol application after ischemia does not interfere with RIPC-induced cardioprotection, whereas administration prior to RIPC abolishes the infarct size reduction after I/R injury in rat hearts (32). Furthermore, a clinical study investigating the delayed protection window of RIPC could demonstrate under propofol anesthesia a protective effect against development of AKI after cardiac surgery (26). Moreover, not only propofol but also sevoflurane may potentially interfere with RIPC-mediated cardioprotection. In a study by Cho et al., the RIPC procedure performed under sevoflurane and remifentanil anesthesia failed to induce cardioprotection (17). The authors employed a model similar to that used in the present study, in which dialyzed RIPC plasma obtained from patients was transferred to isolated rat hearts prior to I/R injury. Interestingly, in this study, a RIPC procedure under propofol and remifentanil anesthesia showed also no cardioprotective effect. However, a recently published meta-analysis of 79 randomized controlled trials showed that the use of volatile anesthetics like sevoflurane had no negative influence on the protective effect of RIPC (18) as the incidence of AKI in patients undergoing cardiac surgery was reduced when they received only volatile anesthetics. Therefore, propofol rather than sevoflurane is more likely to have a confounding effect on the protection by RIPC. For future studies, strict exclusion of propofol or at least RIPC treatment prior to the use of propofol should be preferred.

Another potential confounding factor that could limit the cardioprotective effect of RIPC in this study is the demographic profile of the patient cohort, which consisted exclusively of older male patients (mean age: 57 ± 10 years). A previous study using a similar plasma transfer approach demonstrated that RIPC plasma from young, but not elderly, male volunteers induced cardioprotection in young, male rat hearts. Interestingly, plasma from female volunteers exhibited cardioprotective effects irrespective of whether RIPC was performed (22). Future studies will have to show whether a protective effect can be demonstrated with a younger and female cohort of HTX patients.

Taken together, several factors—including comorbidities, age, sex, and possibly their interaction—can diminish the release or efficacy of humoral cardioprotective signals. Therefore, in the present study, the combination of advanced age, male sex, comorbidities like hypertension, critical illness of the HTX patients and propofol anesthesia could collectively have diminished or masked a cardioprotective potential of the analyzed RIPC plasma.

### 4.1 Limitations

A limitation of this study is the lack of verification that the RIPC procedure performed in HTX patients was indeed successful in inducing a protective response. It is possible that plasma collected during the early window of protection—immediately after the RIPC procedure—may have had greater cardioprotective potential. In such samples, potential confounding influences from the HTX procedure itself and postoperative pharmacological treatments would have been excluded. Additionally, although the patient cohort was homogeneous with respect to the measured confounders, all patients were critically ill and multimorbid. This resulted in diverse preoperative medication regimens and varying severity of the underlying cardiac conditions that necessitated heart transplantation, making it challenging to identify specific inhibitory factors due to potential unmeasured confounders. Another limitation of this study is the method used to determine coronary flow (CF), which was based on weighing the effluent over a defined time period. Although this is one of the two commonly used measurement methods, coronary flow should preferably be measured using a flowmeter, as this would help to minimize potential measurement errors more effectively (33). As with all *in vitro* studies, the findings presented here cannot be directly extrapolated to humans. Thus, a cardioprotective effect of RIPC plasma from HTX patients should further analyzed in *in vivo* models.

## 5 Conclusion

In conclusion, RIPC plasma from HTX patients obtained after surgery and at two time points within the classic delayed protection window has no cardioprotective effect against I/R injury when transferred to rat hearts using the selected experimental setting. The lack of protection is probably due to an inhibition of the protective potential by various confounding factors frequently present in critically ill HTX patients. Additionally, factors related to the study design itself—such as anesthesia—may have further impaired the detection of a protective effect. Therefore, further studies are needed to determine whether a modulated experimental setting, plasma from female or younger HTX patients, tighter control of approved drugs (e.g., exclusion of propofol), or use of plasma obtained directly after RIPC could result in protection after all.

## Data Availability

The raw data supporting the conclusions of this article will be made available by the authors, without undue reservation.
