# Supplementary material for: Transfer of remote ischemic preconditioning plasma from heart transplant patients into isolated perfused rat hearts prior ischemia/reperfusion injury—a translational study of cardioprotection
Source: Front Cardiovasc Med. 2025 Aug 29;12:1631222. doi: 10.3389/fcvm.2025.1631222 (PMC12425904; doi:10.3389/fcvm.2025.1631222)
Supplement: Supplementary file 1 [file Image1.pdf]

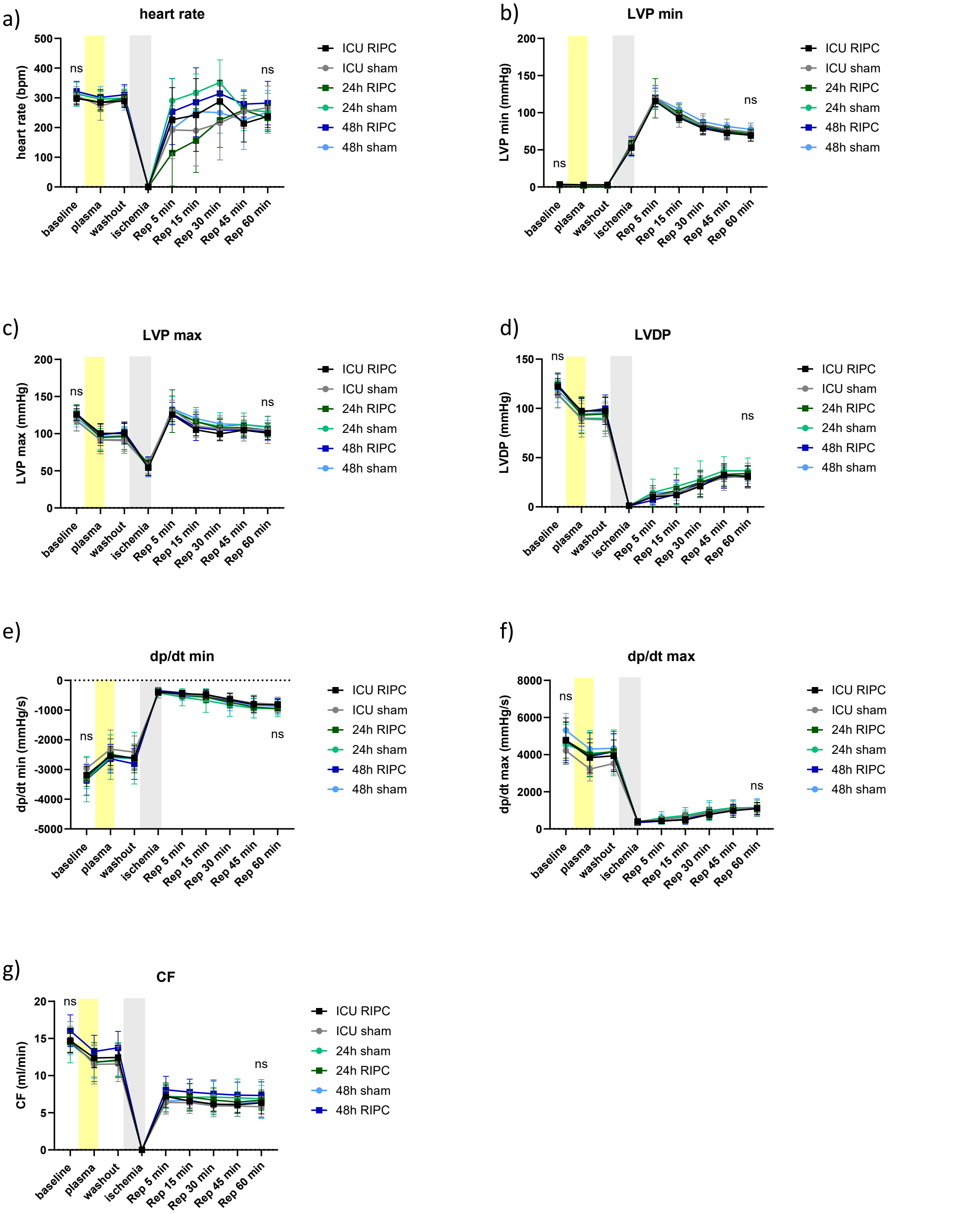

Figure S1 Hemodynamic variables for rat hearts treated with human plasma (yellow box) before ischemia (gray box) and reperfusion (Rep). Plasma was obtained from patients pretreated with a sham or a remote ischemia preconditioning (RIPC) procedure before heart transplantation (HTX) and collected at three time points: on arrival in the intensive care unit (ICU) after HTX or 24 or 48 hours after surgery. a) LVP max (left ventricular systolic pressure); b) LVP min (left ventricular diastolic pressure); c) LVDP (left ventricular developed pressure (LVDP = LVP max - LVP min)); d) heart rate; e) dP/dt max (maximal rate of rise of LVP), f) dP/dt min (minimal rate of rise of LVP); g) coronary perfusion flow (CF). Data are mean +/- SD. Statistical tests were only performed for baseline and at 60 min of reperfusion (Rep 60 min). One-way ANOVA, ns: not significant ($p > 0.05$). $n = 9$.
